# Reactive oxygen species in skin diseases: pathogenic mechanisms and nanomaterial-based therapeutic strategies

**DOI:** 10.3389/fbioe.2026.1797044

**Published:** 2026-04-01

**Authors:** Qi Yan, Yu Zheng, Long Chen, Hongmin Ma, Chao Ding, Xiaoxiao Pang, Tingting Xia, Jingyan Wei, Yinlong Zhang, Guoxin Xu

**Affiliations:** 1 Department of Clinical Laboratory, Zhangjiagang Hospital affiliated to Soochow University, Suzhou, China; 2 Department of Respiratory Medicine, Zhangjiagang Hospital affiliated to Soochow University, Suzhou, China; 3 College of Pharmaceutical Science, Jilin University, Changchun, China; 4 School of Nanoscience and Engineering, University of Chinese Academy of Sciences, Beijing, China

**Keywords:** antioxidants, nanodelivery systems, nanomaterials, ROS, skin disease

## Abstract

Reactive oxygen species (ROS) are inevitable by-products of aerobic metabolism and play a dual role in skin physiology and pathology. At physiological levels, ROS act as essential second messengers regulating cellular signaling and maintaining skin homeostasis. However, excessive ROS accumulation disrupts redox balance, leading to oxidative stress, inflammation, barrier dysfunction, and macromolecular damage, which are closely associated with the pathogenesis of various skin diseases, including psoriasis, atopic dermatitis, pigmentary disorders, photoaging, and skin cancers. In recent years, increasing attention has been directed toward nanomaterial-based strategies for precise ROS regulation, owing to their unique physicochemical properties, such as high surface area, tunable antioxidant activity, and enhanced skin permeability. Compared with conventional antioxidants, nanomaterials, including nanozymes, metal-based nanoparticles, biomacromolecular nanomaterials, and ROS-responsive nanocarriers, exhibit superior stability, targeted delivery capability, and sustained therapeutic efficacy. These nanoplatforms can not only efficiently scavenge excessive ROS but also modulate redox-sensitive signaling pathways, inflammatory responses, and skin barrier repair in a disease-specific manner. This review systematically summarizes the core mechanisms by which ROS contribute to the development of skin diseases, with an emphasis on oxidative stress mediated inflammation, macromolecular damage, and barrier impairment. Furthermore, we comprehensively discuss recent advances in nanomaterial-based therapeutic approaches for ROS regulation, highlighting self-therapeutic nanozymes, biomacromolecular antioxidant materials, and antioxidant-loaded nanodelivery systems. Finally, current challenges and future perspectives for the clinical translation of ROS-targeted nanotherapies in dermatology are discussed, aiming to provide a theoretical basis for the rational design of next-generation nanomedicines for skin disease treatment.

## Graphical Abstract

**Figure:**
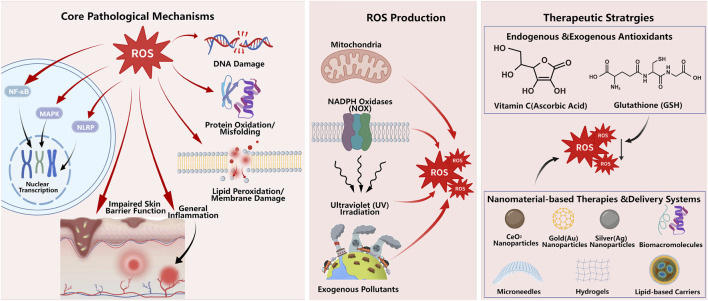

## 1 Introduction

Reactive oxygen species (ROS) are a group of highly reactive, short-lived oxygen-containing molecules that are present in both biological systems and the environment (Spanolios et al., 2024). ROS are inevitable byproduct of normal aerobic metabolism in humans (Liu et al., 2020). Low levels of ROS can regulate important transcription factors (NF-κB/IκB, Nrf2/KEAP1, AP-1, p53, HIF-1) and function as redox signaling molecules in various pathways to maintain cellular homeostasis (Anwar et al., 2024). However, the balance between ROS production and elimination is disrupted by chemical factors like smoking and alcohol consumption (Yang et al., 2022), or physical stimuli like radiation, ultrasound, light, heat, and electric fields (Arfin et al., 2021). The excess ROS can oxidatively damage critical components of signal molecules, cytokines, cell membranes, and cell nuclei, such as proteins, nucleic acids, carbohydrates, and lipids, and meanwhile interfere with cellular processes including apoptosis, migration, differentiation, and proliferation (Anwar et al., 2024; Sauer et al., 2001).

As the largest organ of the human body and the first line of defense against the external environment, the skin can shield the internal organs from damage (Ansary et al., 2021). Nevertheless, continuous exposure to various ROS-generating sources leads to redox imbalance and excessive ROS accumulation. ROS can further cause pathological alterations in skin tissue by attacking biological macromolecules and interfering with cellular structure and function. In recent years, numerous studies have gradually uncovered the core role of ROS in various skin diseases, including inflammatory skin diseases (such as psoriasis, atopic dermatitis), skin aging, and skin tumors. The mechanism of ROS in different skin diseases varies. It mostly involves destroying biological macromolecules, activating signaling pathways linked to inflammation, and impairing the integrity of the epidermal barrier. Treatment approaches based on ROS modulation have been continuously developing in recent years due to its importance in skin disorders. Among these, nanomaterials have garnered a lot of interest because of their unique characteristics. In this review, we focus on summarizing the core mechanisms of ROS-induced skin pathological damage and the treatment strategies relying on nanomaterials, with the aim of providing new insights and theoretical foundations for research of the pathogenesis of skin diseases and clinical treatment.

## 2 Sources of ROS in the skin and initiation of oxidative stress

**FIGURE 1:**
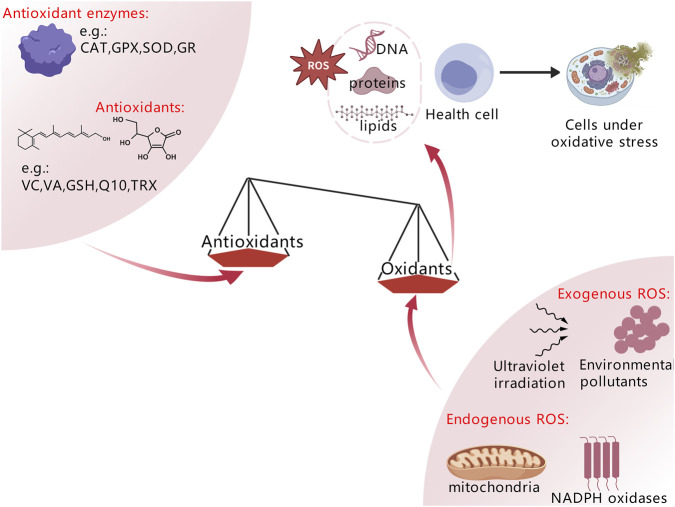
The pathways of ROS production and elimination.

There are two main sources of ROS in the skin: endogenous and exogenous. Endogenous ROS are mostly produced during cellular respiration and energy metabolism, mainly from mitochondria and NADPH oxidases (NOX). Mitochondria are essential oxygen-consuming organelles in the synthesis of ATP in skin cells, where oxygen undergoes electron transfer processes to produce ROS (Wróblewska et al., 2025). NOXs are a family of membrane-bound enzymes expressed in epidermal keratinocytes and skin fibroblasts. When the skin is infected or stimulated by ultraviolet radiation, NOX activity increases significantly, producing large amounts of superoxide anion (O_2_^•−^) in the skin, which induces high cytotoxicity (Figure 1) (Nakai and Tsuruta, 2021). In the exogenous pathway, ultraviolet irradiation is the primary inducer, both medium-wave ultraviolet (UVB) and long-wave ultraviolet (UVA) can activate photosensitive substances in skin cells through photochemical reactions, generating substantial ROS (Wei et al., 2024). Additionally, environmental pollutants, heavy metal ions, chemical agents, and microbial toxins can directly or indirectly induce ROS production in skin cells (Gu et al., 2024; Mitra et al., 2015; Zhao L. et al., 2025).

ROS homeostasis is critical for cell survival and protection against cell damage during signal transduction. The antioxidant defense network, composed of antioxidants such as polyphenols, ascorbic acid, vitamin A, α-lipoic acid, thioredoxin (Trx), glutathione (GSH), melatonin, coenzyme Q, β-carotene, and α-tocopherol, along with antioxidant enzymes including catalase (CAT), glutathione S-transferase (GST), glutathione peroxidase (GPx), superoxide dismutase (SOD), and glutathione reductase (GR) (Ighodaro and Akinloye, 2018; Malinska et al., 2010) can scavenge various types of ROS, achieve ROS detoxification, maintain dynamic balance, and ensure normal cellular physiological activities. However, when endogenous and exogenous stimuli lead to excessive ROS production or impaired antioxidant system function, ROS accumulate in skin tissue, triggering oxidative stress. In addition to directly causing oxidative damage to DNA, proteins, and lipids, which leads to altered cell function, oxidative stress also functions as a second messenger to trigger downstream signaling pathways, control the expression of growth and inflammatory factors, and start cascade reactions (Wei et al., 2024).

## 3 Core pathological mechanisms of ROS-mediated skin diseases

**FIGURE 2:**
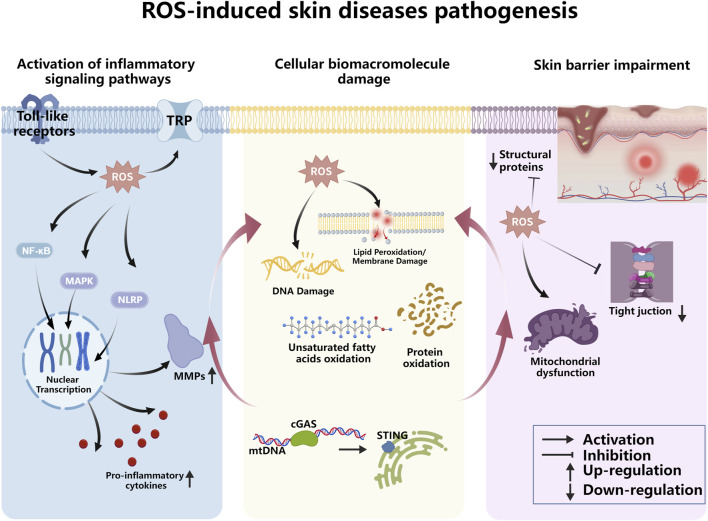
ROS-induced skin diseases pathogenesis.

Reactive oxygen species play complex and critical roles in the occurrence and development of skin diseases (Figure 2), with pathological mechanisms involving multiple levels, including abnormal cell signaling pathways, regulation of inflammatory responses, and direct oxidative damage. The mechanisms of ROS action share commonalities while also exhibiting distinct characteristics across different types of skin diseases.

### 3.1 Activation of inflammation-related signaling pathways in skin diseases

**FIGURE 3:**
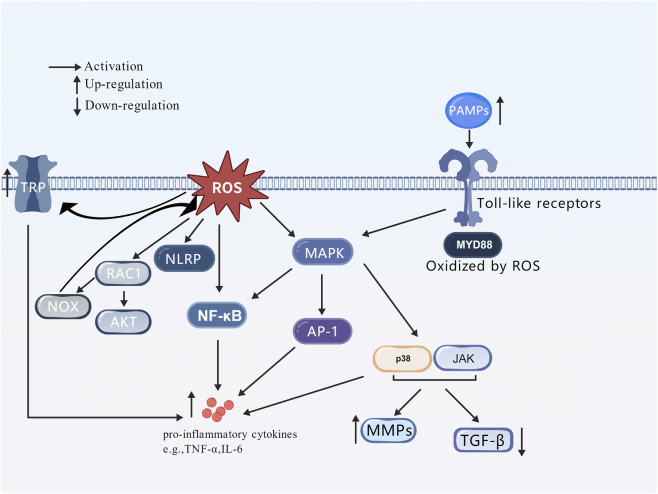
ROS activation of inflammation-related signaling pathways.

ROS are important regulators of inflammatory responses, capable of activating various inflammatory signaling pathways, promoting the secretion and release of inflammatory factors, and forming a chronic inflammatory microenvironment (Figure 3). Nuclear factor κB (NF-κB) is a key transcriptional regulator that controls the expression of multiple genes involved in inflammatory responses. In psoriatic lesions, the NF-κB activity can be upregulated by ROS (Bi et al., 2023), thereby promoting the expression of pro-inflammatory cytokines such as tumor necrosis factor-α (TNF-α), interleukin-6 (IL-6), and interleukin-17 (IL-17). These cytokines not only regulate the functions of dendritic cells and Th7 cells to maintain the local inflammatory environment but also stimulate the excessive proliferation of keratinocytes, leading to the characteristic epidermal thickening and abnormal differentiation of psoriasis, and forming an “inflammation-proliferation” vicious cycle. ROS can also activate the NLRP3 inflammasome, thereby promoting the secretion of inflammatory factors such as IL-18 and IL-1β, further exacerbating the body’s inflammatory response and damaging the endothelial vascular barrier, resulting in skin vascular abnormalities, which is a hallmark of psoriasis (Yue et al., 2025). Cyclooxygenase-2 (COX-2) is a key enzyme in the cyclooxygenase family, primarily involved in inflammatory responses and prostaglandin synthesis. ROS can increase COX-2 expression to promote the synthesis of prostaglandin E2 (PGE2), which acts as an inflammatory mediator to participate in vasodilation and inflammatory cell infiltration in psoriatic lesions (Wilcox et al., 2019). As the main exogenous ROS-inducing factor, ultraviolet radiation induced ROS can activate the MAPK pathway and its downstream factors NF-κB and AP-1, thereby regulating the release of inflammatory cytokines such as IL-1β, IL-6, and TNF-α. After activation, two important downstream factors of the MAPK signaling pathway, p38 and JNK can enter the nucleus, upregulate the expression of related inflammatory cytokines, induce the expression of matrix metalloproteinases (MMPs), and downregulate the expression of TGF-β, reducing the synthesis of new collagen, thus playing a crucial role in ultraviolet-mediated photoaging (Wei et al., 2024). ROS produced during photodamage can activate inflammasomes; among these, NLRP1 is more highly expressed in human skin compared to other NLRs. Mutations in the NLRP1 gene are the genetic etiological basis of skin diseases and increase the risk of skin cancer (Awad et al., 2018). Studies have demonstrated that NLRP1 activation in tumor cells can activate caspase-2 and caspase-9, thereby promoting tumorigenesis (Zhai et al., 2017). Conversely, suppression of inflammasome assembly and IL-1β expression has been shown to inhibit cancer cell proliferation. Consistent with this, murine models harboring genetic defects in inflammasome components exhibit reduced tumor progression, suggesting a pro-tumorigenic role for inflammasome signaling (Moossavi et al., 2018). Studies have shown that patients with atopic dermatitis have defective skin barrier function, and external stimuli can easily induce ROS production. ROS can participate in the activation of mast cells, upregulate the expression of TRPA1 (a member of the transient receptor potential (TRP) ion channel superfamily involved in inflammatory responses), and promote the release of inflammatory factors IL-6 and IL-23 (Hu et al., 2025). In melanoma, ROS can trigger melanoma invasion by enhancing the expression of Rac family small GTPase 1 (RAC1) and then activating downstream signals of the AKT pathway (Liu-Smith et al., 2014); meanwhile, RAC1 is a key activator of NOX enzymes. Activated RAC1 binds to the C-terminus of NOX1, activating NOX1 and inducing ROS production, which is prone to forming a vicious cycle (Liu-Smith et al., 2014). The expression of Toll-like receptors (TLRs) can initiate inflammatory responses, and their expression has been detected in skin keratinocytes, particularly TLR2 and TLR4. These receptors can recognize pathogen-associated molecular patterns (PAMPs) such as LTA and LPS. Activated TLR receptors can trigger downstream signaling cascades, including the NF-κB and MAPK pathways, thereby releasing pro-inflammatory cytokines and chemokines such as IL-6, IL-1β, and TNF-α. Excessive or prolonged TLR signaling can lead to chronic inflammation and tissue damage, thereby triggering a series of skin diseases (Haroon and Kang, 2025). ROS can enhance Toll-like receptor signaling through two mechanisms: inducing damaged cells to release high mobility group box 1 (HMGB1) damage-associated molecular patterns to activate Toll-like receptors (Gill et al., 2010); and directly oxidatively modifying the intracellular domain of Toll-like receptors or downstream adapter proteins (e.g., myeloid differentiation factor 88, MyD88) to increase their binding affinity to ligands (Stottmeier and Dick, 2016).

### 3.2 ROS-induced damage to cellular biological macromolecules

**FIGURE 4:**
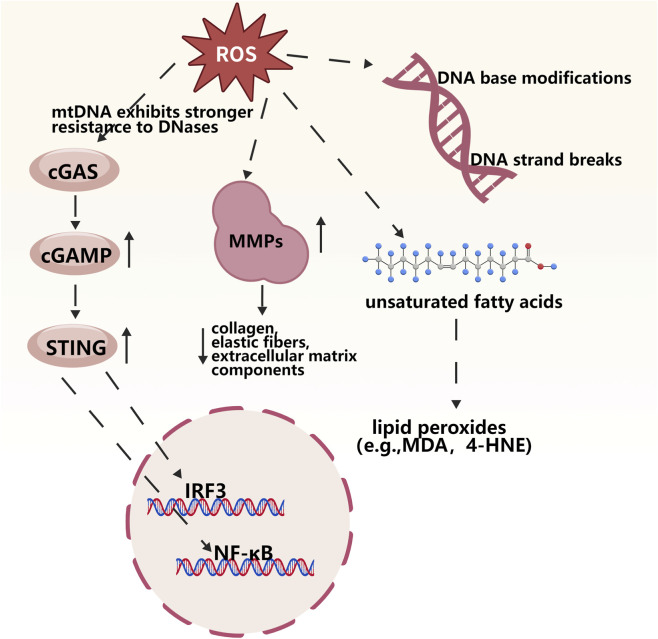
Overview of how reactive oxygen species (ROS) participate in the pathological processes of skin diseases by directly or indirectly attacking intracellular biological macromolecules.

ROS possess strong oxidizing properties and can directly or indirectly attack intracellular biological macromolecules, disrupting cellular structure and function, thereby mediating cell apoptosis or abnormal proliferation and participating in the pathological process of skin diseases (Figure 4). ROS induced by ultraviolet radiation can cause DNA base modifications (e.g., 8-oxoguanine formation), single-strand and double-strand breaks, and cross-linking damage. If these damages are not repaired in a timely manner, they can lead to the activation of proto-oncogenes or inactivation of tumor suppressor genes; for example, p53 mutations are very common in ultraviolet-related squamous cell carcinoma (Liu et al., 2023; Xian et al., 2019). cGAS belongs to the nucleotide cyclase family and serves as a sensor for double-stranded DNA (dsDNA). Both mitochondrial DNA (mtDNA) and nuclear DNA (nDNA) can be recognized by cGAS. Upon binding to dsDNA, cGAS transitions from an inactive to an active state and generates cyclic GMP-AMP (cGAMP). Subsequently, cGAMP is detected by the STING protein on the endoplasmic reticulum (ER) membrane, and activates it through conformational changes, thereby triggering the NF-κB and interferon regulatory factor 3 (IRF3) signaling pathways and promoting the secretion of pro-inflammatory cytokines and type I interferons. mtDNA affected by oxidative stress exhibits stronger resistance to deoxyribonucleases (DNases), which promotes cGAS recognition and STING activation (Waseem et al., 2025). Skin cell membranes are rich in unsaturated fatty acids and are highly susceptible to ROS-induced peroxidative damage. ROS attack triggers a chain reaction, generating lipid peroxides such as malondialdehyde (MDA) and 4-hydroxynonenal (4-HNE), which destroy the integrity of cell membranes, reduce the fluidity of the lipid bilayer, and impair cell permeability (Li Pomi et al., 2025; Liu et al., 2023; Prasad et al., 2023). Matrix metalloproteinases (MMPs), especially MMP-1, MMP-3, and MMP-9, can degrade collagen, elastic fibers, and other extracellular matrix components (Kim et al., 2019). ROS can upregulate the expression of MMP proteins, thereby indirectly damaging protein molecules and promoting skin aging.

### 3.3 ROS-mediated impairment of skin barrier function

**FIGURE 5:**
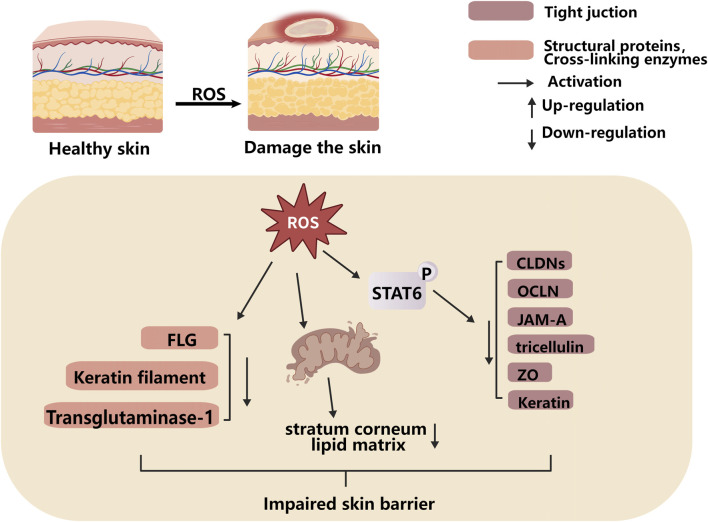
ROS affects skin barrier function by down-regulating the expression of protein related to the skin barrier.

The stratum corneum, tight junctions, and extracellular matrix make up the skin barrier, which is an essential element for preserving skin homeostasis. ROS damage skin barrier function through a number of interconnected mechanisms (Figure 5). The expression levels of cross-linking enzymes and structural proteins produced by cells—such as keratin laminin, filaggrin (FLG), transglutaminase-1, and keratin filament—that are related to skin barrier function are downregulated under the action of ROS, thereby impairing skin barrier function (Dusabimana et al., 2024). Tight junctions seal the paracellular spaces of keratinocytes to form the epidermal barrier; molecules involved in tight junctions include claudins (CLDNs), occludin (OCLN), JAM-A, tricellulin, zonula occludens (ZO), and keratin. ROS production can maintain the phosphorylated state of STAT6, aggravate mitochondrial apoptosis, thereby affecting the expression of tight junction proteins and severely impairing skin barrier function (Wang et al., 2023; Wang X. et al., 2024). By taking part in cellular metabolism, energy production, and oxidative stress reduction, mitochondria serve important physiological functions of the skin cell division, repair, and regeneration require energy, which is inseparable from ATP generated by mitochondria. Mitochondria can also indirectly promote the production of the stratum corneum lipid matrix, which plays a critical role in skin barrier function and preventing water loss. Excessive ROS are prone to cause mitochondrial dysfunction, leading to impaired skin barrier development and abnormal skin defects (Ahmad Jamil and Abdul Karim, 2025).

## 4 Therapeutic strategies based on ROS regulation

**FIGURE 6:**
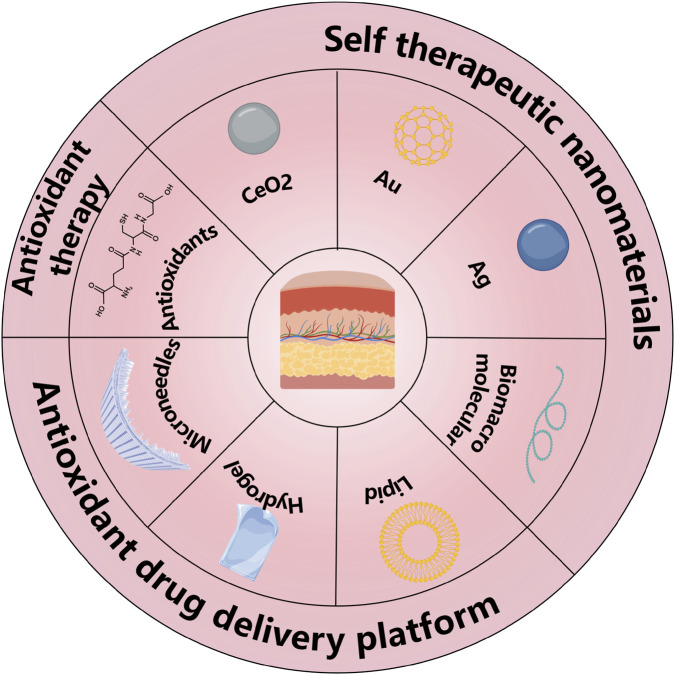
Schematic illustration of traditional antioxidants and different nanoparticle/nanocarrier-based therapeutic modalities for ROS-related skin diseases.

With the deepening understanding of the role of ROS in skin diseases, various therapeutic strategies targeting ROS regulation have been developed. These strategies include the optimized application of traditional antioxidants and the design of novel nano-drug delivery systems (Figure 6). Each of these therapeutic strategies has unique characteristics, targeting the features of ROS abnormalities in different skin diseases and providing more options for clinical treatment.

### 4.1 Antioxidant therapy

Antioxidants are direct strategy to neutralize ROS and reduce oxidative stress, and play an important role in the treatment of skin diseases. Antioxidants can be classified as either endogenous (such as glutathione, coenzyme Q10, and thioredoxin) or exogenous (such as vitamin E, vitamin C, carotenoids, and polyphenolic substances) based on their sources and characteristics. These antioxidants exert their effects through various mechanisms, including direct scavenging of free radicals, interrupting free radical chain reactions, chelating transition metal ions, and upregulating the endogenous antioxidant defense system.

In the treatment of skin diseases, several representative antioxidants have shown significant efficacy. As a water-soluble antioxidant, vitamin C can not only directly scavenge ROS but also increase the content of antioxidant enzymes in the body, forming a synergistic antioxidant network (Wu et al., 2025). Additionally, vitamin C is a cofactor of prolyl hydroxylase, participating in collagen synthesis, which is crucial for maintaining skin barrier function and promoting wound healing (Pullar et al., 2017). The level of vitamin C in the skin decreases with age, and exogenous supplementation can improve signs of photoaging (Shindo et al., 1994).

Quercetin, a natural flavonoid compound, exerts anti-inflammatory and antioxidant effects through multiple mechanisms, including downregulating pro-inflammatory factors such as TNF-α and IL-1α (Bureau et al., 2008), blocking the COX-2 signaling pathway, and directly neutralizing ROS (Lee et al., 2010). Quercetin shows promising prospects in the prevention and treatment of skin cancer, as it can inhibit the proliferation of melanoma cells and induce apoptosis (Okselni et al., 2025). However, traditional quercetin has poor water solubility, low skin permeability (<5%), and a severe first-pass effect, which limit its clinical application (Potiguara de Moraes et al., 2025).

Epigallocatechin gallate (EGCG), the main active component of green tea polyphenols, possesses potent antioxidant and anti-inflammatory properties. Studies have shown that EGCG can significantly reduce the content of ROS in mitochondria and delay cellular senescence (Park et al., 2025); furthermore, EGCG can alleviate aging-related phenotypes by increasing collagen expression. In the treatment of psoriasis, EGCG can relieve the inflammatory environment by inhibiting the secretion of pro-inflammatory cytokines IL-1β and TNF-α associated with the pathogenesis of psoriasis (Chamcheu et al., 2018).

Coenzyme Q10 is an endogenous antioxidant found in bovine heart mitochondria, playing a key role in mitochondrial electron transport and antioxidant defense, and is a critical factor in maintaining health. Coenzyme Q10 can reduce UVB-induced skin damage in mice, exerting a good protective effect on the epidermis and maintaining its integrity; it can inhibit the production of MMP-1 to protect skin collagen; in addition, coenzyme Q10 can reduce the increase in MDA content caused by ultraviolet irradiation and enhance the levels of antioxidant enzymes in the body. With age, the level of coenzyme Q10 in the skin decreases, and exogenous supplementation can improve signs of skin aging (Wu H. et al., 2020).

Despite the theoretical appeal of antioxidant therapy, its clinical application faces several challenges. Many antioxidants have poor stability and are easily oxidized and inactivated; their skin permeability is low, making it difficult to reach effective concentrations; systemic administration is hindered by issues such as the first-pass effect and rapid clearance. These limitations have prompted researchers to develop more advanced delivery systems to enhance the therapeutic efficacy of antioxidants.

### 4.2 Nanomaterial-based therapy

The application of nanotechnology in the field of drug delivery has brought revolutionary changes to the treatment of ROS-related skin diseases. Nanomaterials have attracted attention due to their characteristics, such as large specific surface area, high surface activity, and strong modifiability, they can effectively overcome the limitations of traditional drugs. Nanomaterial-based drug delivery systems can successfully overcome the drawbacks of conventional antioxidants while also improving medication stability, skin penetration, targeted delivery, and controlled release. According to the characteristics of the disease and the treatment requirements, different types of nanomaterial treatment methods can be designed.

#### 4.2.1 Self-therapeutic nanomaterials

##### 4.2.1.1 Metal nanozymes

CeO_2_ nanoparticles are a common kind of nano-antioxidant that can efficiently remove excessive ROS generated by pathogenic cells and restore redox equilibrium because they have CAT-like and SOD-like properties (Zhou et al., 2025) (see Table 1). The effective antioxidant capacity of CeO_2_ nanoparticles enables them to exhibit excellent therapeutic effects in various ROS-related diseases (Xu et al., 2022). Tarnuzzer et al. conducted an investigation into the antioxidant properties of CeO_2_ nanoparticles to evaluate their protective effects on cells. The results demonstrated that a concentration as low as 10 nM of CeO_2_ nanoparticles could provide nearly complete cellular protection against radiation-induced damage (Tarnuzzer et al., 2005). In addition to using CeO_2_ nanoparticles on their own, scientists have modified them in a number of ways to increase their effectiveness. Oxidative stress induced by ROS plays a crucial role in the pathogenesis of psoriasis. Wu L. et al. modified CeO_2_ with synthetic β-cyclodextrins (β-CDs) and applied it (1 mg) to the IMQ-induced psoriasis model, it effectively reduced the TNF-α content in the psoriasis model and demonstrated excellent therapeutic effects (Wu L. et al., 2020). CeO_2_ (100 mM) can effectively reduce the increase of ROS caused by ultraviolet radiation in the ultraviolet damage model and significantly increase the level of antioxidant enzymes in cells, thereby resisting the photodamage induced by ultraviolet rays (Ribeiro et al., 2020); The water gel encapsulated CeO_2_ (Ce@Col gels) endows it with higher stability, improves the ROS clearance ability, further reduces the level of MMP-1, and 200 μg Ce@Col gels can effectively alleviate DNA damage and collagen degradation and the epidermal thickening and skin wrinkling caused by ultraviolet radiation (Kim et al., 2025). Skin regeneration and wound healing are hampered by excessive ROS production. Based on the cerium oxide nano-composite hydrogel, researchers applied it to the mouse full-thickness skin injury model, and the results showed that the composite hydrogel (200 µL) could significantly accelerate the wound healing speed and shorten the healing time (Gong et al., 2022). Compared with CeO_2_ nanoparticles and nanorods, porous CeO_2_ nanorods have more surface catalytic centers, thus presenting higher catalytic activity. In the atopic dermatitis model, it can regulate oxidative stress, reduce the secretion of inflammatory factors, effectively inhibit the activation of the NF-κB pathway, and alleviate the symptoms of atopic dermatitis (Bai et al., 2025). The application of CeO_2_ in skin diseases suggests that further exploration of it will be helpful for the development of drugs for skin injuries.

**TABLE 1:** Nanomaterials with self-healing properties are used for the treatment of skin diseases.

Nanoparticles	Mechanism	Related diseases	References
CeO_2_	Inhibit cell apoptosis	Radiation-induced damage	Tarnuzzer et al. (2005)
​	Eliminate superoxide anion and H_2_O_2_	Psoriasis	Wu L. et al. (2020)
​	Reduce ROS and increase the level of antioxidant enzymes	Photodamage	Ribeiro et al. (2020)
​	Reduce the level of MMP-1	Photodamage	Kim et al. (2025)
​	​	Skin wound	Gong et al. (2022)
​	Inhibit the activation of the NF-κB pathway	Atopic dermatitis	Bai et al. (2025)
Gold	Downregulate inflammatory genes	Psoriasis	Han et al. (2021)
​	Reduce the generation of pro-inflammatory	Chronic skin inflammation	Zhao et al. (2024)
​	Reduce ROS	Photodamage	Li et al. (2025)
Silver	Reduce ROS and inhibit the activation of macrophages by NF-κB	Psoriasis	Crisan et al. (2018)
​	Repair large-scale DNA damage	Photodamage	Tyagi et al. (2016)
​	Resist the high ROS environment	Atopic dermatitis	Pan et al. (2025)
Chitosan	Reduce the expression of pro-inflammatory factors and increase the expression of anti-inflammatory factors	Skin wound	He et al. (2024)
​	Stimulated fibroblast migration, proliferation, and angiogenesis	Skin wound	Zhang et al. (2025)
​	Balance inflammatory responses	Burn	Xiao et al. (2025)
​	​	Photodamage	Heydari et al. (2025)
Peptide hydrogel	Remove excessive ROS and eliminate inflammation	Radiation-induced damage	Hao et al. (2023)

Excellent biocompatibility, colloidal stability, and water solubility characterize gold nanoparticles, which can efficiently remove a variety of ROS, such as superoxide anions and hydroxyl radicals (·OH), and have some enzyme-like properties (Xu et al., 2022; Zhang et al., 2021). The antioxidant and anti-inflammatory properties of gold nanoparticles make them show good therapeutic potential in various diseases. According to much research, the surface modification of gold nanoparticles has variable biological consequences (Xu et al., 2022). Modifying gold nanoparticles with 30% octadecyl chains (∼3 µM) can reduce the expression of genes linked to inflammation and epidermal thickness in IMQ-induced psoriasis model mice without the use of any biological or chemical medications (Han et al., 2021). The nitrogen-containing cyclopropane monoethanolamine-functionalized gold nanoparticles (Au-MEA NPs) prepared by the ligand exchange method using citric acid-terminated gold nanoparticles (Au-CA NPs) can effectively alleviate psoriasis-like and rosacea-like chronic skin inflammation by promoting SOD activity and inhibiting MMP9 activity, thereby reducing the generation of pro-inflammatory mediators in keratinocytes (Zhao et al., 2024). Additionally, the effectiveness of other medications can be increased by loading them onto gold nanoparticles. By loading gold nanoparticles into mesoporous polydopamine nanospheres (mPDA) and constructing Au@mPDA nanoparticles containing estradiol (E2), the ROS content is further reduced, and the ability of E2 to alleviate oxidative stress and inflammatory responses is enhanced, significantly enhancing its therapeutic effect on skin aging (Li et al., 2025). Any *in vivo* application of nanoparticles requires a comprehensive understanding of their kinetic characteristics and toxicological features. Although gold nanoparticles can accumulate in the liver and spleen, they do not cause abnormal behavior in experimental animals. Their favorable safety and therapeutic qualities attest to their suitability for upcoming biological uses (Lopes et al., 2023).

Silver nanoparticles have drawn interest since numerous studies have demonstrated their potential for immunological regulation and ROS control activities (Xu et al., 2022; Xu et al., 2020; Yang et al., 2021). The therapeutic effects in various skin diseases have also been explored, and the specific mechanisms vary depending on the disease. The results of *in vitro* experiments (1.9 μg/mL) and studies on human psoriasis (2%) show that silver nanoparticles can effectively inhibit the activation of macrophages by NF-κB, reduce the release of ROS and pro-inflammatory factors, and ultimately lead to the remission of psoriasis symptoms (Crisan et al., 2018). Silver nanoparticles (2 μg/mL) can also repair large-scale DNA damage through the nucleotide excision repair (NER) mechanism, thereby protecting HaCaT cells from UVB-induced DNA damage. At the same time, the activities of SOD/CAT/GPx in the cells increase under the action of Ag-NPs, providing ROS protection for skin cells (Tyagi et al., 2016). Atopic dermatitis (AD) is a common chronic inflammatory skin disease caused by immune imbalance, oxidative stress, and bacterial invasion. The accumulation of ROS can aggravate the symptoms of AD. Pan et al. designed a hydrogel microneedle patch, which is composed of hyaluronic acid modified with benzhydrylamine (HA-PBA) as the matrix and tannic acid (TA)/silver nanoparticles (TA/Ag NPs) as the crosslinking agent. This material can effectively resist the high ROS environment inside and outside cells and exhibits strong antioxidant capacity, at the same time, it can also reduce the level of TNF-α and effectively alleviate the symptoms of AD (Pan et al., 2025). The research results indicate that appropriate silver nanoparticles can be designed for anti-inflammatory and ROS clearance effects to combat various skin diseases.

##### 4.2.1.2 Biomacromolecular nanomaterials

**FIGURE 7:**
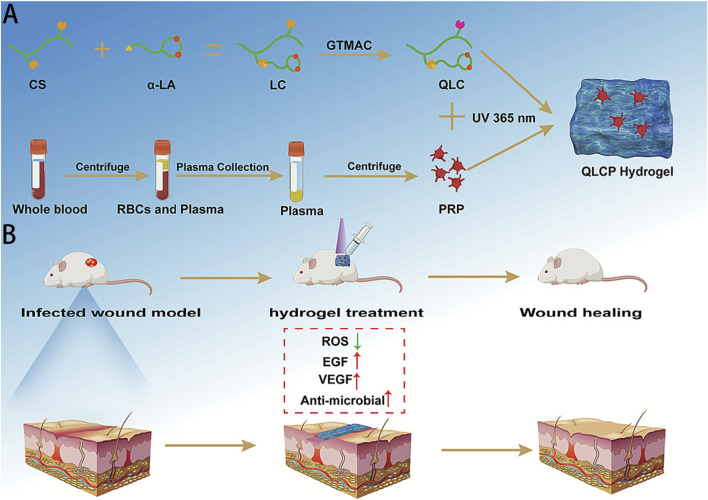
**(A)** The fabrication and composition of QLCP hydrogel, and **(B)** the application of QLCP hydrogel for treating infected wounds. Reprinted with permission from ref. (Zhang et al., 2025).

It is worth noting that some biomacromolecules, such as polysaccharide-based nanoparticles and protein nanoparticles, can also eliminate ROS through their inherent reducing residues. Their inherent antioxidant, anti-inflammatory, immunomodulatory, wound healing promotion, and moisturizing properties make them have special application value in the fields of skin care and tissue repair (Cheong et al., 2025). Chitosan is a biopolymer obtained by partial deacetylation of chitin. It is usually combined with other materials for application. Guo et al. synthesized a hydrogel by one-step synthesis using quaternized chitosan (QCS), tannic acid (TA), and trivalent iron [Fe(III)], which can effectively eliminate free radicals and has significant effects on wound closure and promoting the healing process (Guo et al., 2022); Based on hydrogen bonds and Schiff base interactions, carboxymethyl chitosan (CMCS), oxidized dextran (Odex), and oligomeric proanthocyanidins (OPC) have formed a novel water gel with good injectability, self-repairing properties, and adhesiveness through cross-linking. Their potent antibacterial activity enables them to inactivate almost all *E. coli* and *Staphylococcus aureus* within 2 h, reducing the possibility of wound infection. At the same time, by reducing pro-inflammatory factors and increasing anti-inflammatory factors such as IL-10 and CD206 expression, they promote wound healing (He et al., 2024); In the most recent study, Zhang et al. dispersed platelet-rich-plasma (PRP) into QLC, which based on CS modified with α-lipoic acid and GTMAC, to create a new hydrogel QLCP (Figure 7). This design gave CS improved antibacterial and ROS scavenging capabilities in addition to increasing its hydrophilicity. The long-term release of bioactive growth factors made possible by the addition of PRP successfully stimulated fibroblast migration, proliferation, and angiogenesis. It greatly increased the rate at which wounds healed in the skin wound model (Zhang et al., 2025). Based on the acid-responsive hydrogel of carboxymethyl chitosan (CMCS), the intelligent regulation of the ROS-macrophage axis through the Schiff base reaction enables precise management of the wound healing process, effectively promoting the healing of infected burn wounds (Xiao et al., 2025). Recently, researchers have also prepared two new sunscreen hydrogels by combining chitosan with 2,4-dihydroxybenzaldehyde (with or without vanillin) through Schiff base reactions and hydrogen bond interactions. These hydrogels exhibit excellent ultraviolet blocking ability in both UVA and UVB bands and have significant antioxidant potential, effectively inhibiting epidermal thickening caused by ultraviolet radiation (Heydari et al., 2025). Chemotherapy is one of the main methods of cancer treatment, which often causes severe skin damage. Recently, Hao et al. designed a heparin-like peptide hydrogel (K16, KYKYEYEYAGEGDSS-4Sa) composed of a heparin-functional polypeptide (AGEGDSS-4Sa) and a polypeptide with antioxidant and self-assembly properties (KYKYEYEY) through a one-step solid-phase synthesis method, which proved to have good application potential in the field of radiation-induced skin injury repair. K16 can effectively remove excessive ROS. The anionic sulfate group on the 4-sulfonated benzeneacetic acid at the peptide terminus of K16 can form a complex with positively charged inflammatory cytokines, adsorbing more inflammatory cytokines and eliminating inflammation, thereby promoting the healing of radiation-induced skin damage (Hao et al., 2023).

#### 4.2.2 Natural antioxidant drug delivery platform based on nanomaterials

The application of endogenous or exogenous antioxidants is limited by their low permeability and poor stability, despite the fact that they can effectively remove ROS and reduce the evolution of numerous oxidative stress-related illnesses. Nanoparticle technology has shown great potential in converting poorly absorbed and physiologically unstable compounds into effective therapeutic agents (Han et al., 2024) (see Table 2). Whether they are natural compounds, small molecule antioxidants, or large molecule antioxidant enzymes, when modified with nanomaterials, they all exhibit higher cell uptake rates and stability.

**TABLE 2:** Nano systems for delivering antioxidants and their applications in skin diseases.

Delivery platform	Antioxidant in the package	Related diseases	References
Microneedle	EGCG	Psoriasis	Bi et al. (2023)
​	Curcumin	Psoriasis	Xu et al. (2025)
​	Quercetin	Skin cancer	Fang et al. (2025)
Nanoparticles	EGCG	Radiation-induced damage	Han et al. (2024)
​	Quercetin	Skin cancer	Caddeo et al. (2016); Chitkara et al. (2022); Kapare et al. (2024)
​	Flavonoids	Photodamage	Gollavilli et al. (2020)
​	Coenzyme Q10	Photodamage	Pegoraro et al. (2017); Schwarz et al. (2013); Yue et al. (2010)
Nanocomposite materials	Curcumin	Skin wound	Zhao Y. et al. (2025)
​	Catalase	Photodamage	Yan et al. (2024)
Nanoemulsion	Flavonoids	Photodamage	Back et al. (2018)

In recent years, natural compounds such as EGCG, curcumin, and flavonoids have been used in various preclinical and clinical studies due to their excellent reducing properties. However, they still face challenges such as cell uptake obstruction. Nanomaterial delivery systems can effectively solve this problem. Recently, a separable H_2_O_2_-responsive gel microneedle patch containing methotrexate (MTX) and EGCG has been developed and used for the combined treatment of psoriasis. The needle tip of this microneedle is formed by cross-linking hyaluronic acid with side-chain-modified benzylboronic acid and EGCG through dynamic covalent bonds, and it has H_2_O_2_-responsive properties. After the microneedle is inserted into the psoriatic skin, the needle tip separates from the base, and MTX is rapidly released from the swollen gel pores, promptly inhibiting keratinocyte proliferation, while EGCG breaks the bond in response to the high ROS environment in the skin and releases in an intelligent and controlled manner, effectively inhibiting the NF-κB inflammatory pathway for a long time (Bi et al., 2023). Han et al. created EGCG modified with polyethylene glycol and then self-assembled it to form EGCG nanoparticles, which showed improved cell absorption capacity and a higher ROS clearance impact. It (25 mg/kg) was able to effectively reduce the production of pro-inflammatory factors (IL-6/TNF-α) and symptoms like radiation-induced skin edema (Han et al., 2024). The carbon nanocomposite materials prepared using curcumin and zinc acetate as raw materials not only retain the antibacterial and anti-inflammatory properties of curcumin, but also improve its bioavailability, promoting the healing of infected wounds (Zhao Y. et al., 2025); gel, with its biocompatibility and biodegradability, stands out among the water gel materials for skin repair. Combining the unique advantages of curcumin and gel nanoparticles, researchers have prepared a curcumin-loaded nanocomposite hydrogel dressing with both antibacterial and sustained-release drug properties, which significantly improves wound closure, healing, and vascularization (Fu et al., 2024); Although curcumin is effective in treating psoriasis with low adverse reactions, its low water solubility and poor stability reduce its bioavailability (Zhang et al., 2022); Microneedles can administer drugs effectively and conveniently, penetrate the epidermal barrier, while accomplishing accurate targeting. Xu et al. constructed a transdermal nano-microneedle delivery system based on curcumin and applied it to the IMQ-induced psoriasis mouse model (∼10 μg/mL), significantly reducing the levels of pro-inflammatory factors IL-17, IL-22, IL-23, and TNF-α and the thickness of the epidermis, effectively alleviating psoriasis-related symptoms (Xu et al., 2025). Quercetin, as a natural polyphenol, has anti-inflammatory, anti-tumor, and antioxidant properties, making it an ideal choice for biomedical applications. However, quercetin’s research and use are constrained by its instability, poor water solubility, quick metabolism, and inadequate targeted specificity. These issues can be effectively resolved by quercetin nanoparticles and nanoemulsions, which also exhibit improved therapeutic effects due to their increased antioxidant capacity in a variety of skin conditions (Caddeo et al., 2016; Chitkara et al., 2022; Kapare et al., 2024). Recently, Fang et al. prepared a new multifunctional patch based on quercetin nanoparticles and multifunctional microneedles, which not only enhanced its antibacterial and free radical scavenging capabilities but also achieved sustained drug release, effectively inhibiting melanoma recurrence and reducing the levels of pro-inflammatory factors, then promoting skin regeneration (Fang et al., 2025). Additionally, flavonoids with antioxidant activity, such as naringin and soybean extract rich in isoflavones, after being modified by nanomaterials, were endowed with stronger skin permeability, thereby better protecting against ultraviolet-induced skin damage (Back et al., 2018; Gollavilli et al., 2020).

Although antioxidants have a strong ability to remove ROS, the poor stability and difficulty in penetrating the cell membrane limit their clinical development and application. Currently, there are numerous studies on the modification of antioxidant nanoparticles, which further promotes the development of antioxidants (Al-Smadi et al., 2025; Dias and Estevinho, 2025; Lu et al., 2025). Vitamin C, as a natural antioxidant, shows strong clinical application value in various diseases. The vitamin C modified by nanomaterials, to some extent, overcomes its inherent defects, increases the intracellular drug concentration and ROS clearance rate, and has higher bioavailability (Lu et al., 2023). Nanoparticles rich in vitamin E have been endowed with stronger skin permeability, which can better resist oxidative stress and promote the proliferation of skin fibroblasts, and have broad application prospects in skincare, tissue regeneration and other related fields (Sheng et al., 2013; Vaz et al., 2019). Coenzyme Q10 is a potent antioxidant in plasma. Compared with free coenzyme Q10, its delivery using nano-liposomes can enable it to penetrate deeper into the dermis and exert stronger antioxidant performance, further enhancing the activity of intracellular antioxidant enzymes and reducing intracellular ROS content, effectively alleviating skin damage induced by ultraviolet rays (Pegoraro et al., 2017; Schwarz et al., 2013; Yue et al., 2010).

Catalase (CAT) is an endogenous antioxidant that can efficiently scavenge H_2_O_2_. Nevertheless, its high molecular weight limits its antioxidant function by preventing it from penetrating cells. To order to solve this problem, researchers fused chitosan (CS) with N-succinimidyl S-acetylthioacetate (SNAC) to obtain SNAC-substituted chitosan (SCS), which can self-assemble with catalase (CAT) to form stable nanocomplexes, showing excellent therapeutic and protective effects against cell damage caused by ultraviolet irradiation. SCS-CAT enters cells through paracellular pathways, transcellular pathways, and skin appendage pathways; it can not only downregulate inflammatory factors such as TNF-α, IL-6, and IL-1β but also upregulate IL-10 (a cytokine believed to promote sterile tissue repair) to reduce oxidative stress, thereby helping to alleviate dermatitis symptoms. It can also resist photoaging by reducing skin cell apoptosis and regulating collagen synthesis through increasing TGF-β (Yan et al., 2024). This research promotes the development and utilization of macromolecular protein drugs. Nano materials have significantly overcome the inherent drawbacks of antioxidants and greatly advanced the development of clinical drugs.

## 5 Challenges and future prospects

Research on ROS regulation-based treatments for skin conditions has advanced significantly (Bi et al., 2023; Yan et al., 2024), however there are still many obstacles and unsolved problems in this area. From basic research to clinical translation, from single treatment to comprehensive intervention, and from disease control to prevention of recurrence, all links require further exploration and improvement. Meanwhile, the treatment of ROS-related skin illnesses has brought forth previously unheard-of development potential due to the ongoing development of new technologies and materials as well as the strengthening of interdisciplinary integration.

The skin has a special barrier structure, especially the stratum corneum, which makes it difficult for many drugs to reach effective therapeutic concentrations. Although new technologies such as microneedles and nano-carriers have improved skin permeability, achieving deeper targeting (e.g., hair follicles, sweat glands, deep dermis) remains challenging (Lee, 2025). Additionally, most existing systems rely on the inherent high ROS environment at the lesion site to achieve targeted release (Yan et al., 2024), but this passive targeting strategy may have limited efficacy in the early or mild stages of the disease. The development of active targeting systems (e.g., antibody or peptide-guided) may help address this issue but faces new challenges such as high cost and poor stability.

There are differences in the sources, types, and mechanisms of ROS across different skin diseases; even within the same disease, ROS characteristics may vary among different patients or disease stages. For example, the increase in hydrogen peroxide caused by NOX4 mutations in psoriasis and the burst of superoxide anions coupled with mechanical stimulation in atopic dermatitis require different intervention strategies (Wang S. et al., 2024). Current treatments mostly adopt a “one-size-fits-all” approach, lacking the ability for individualized adjustment. Establishing a precise classification system based on patients’ molecular characteristics is a prerequisite for personalized treatment, but research on related biomarkers is still insufficient.

Long-term safety and drug resistance issues cannot be ignored. Chronic skin diseases require long-term treatment, but there is limited safety data on the long-term use of existing antioxidants or nanomaterials. Certain antioxidants may exhibit pro-oxidative activity at high concentrations or under specific conditions (e.g., lipoic acid, vitamin C). The biocompatibility, toxicity of degradation products, and immunogenicity of nanomaterials also require more comprehensive evaluation.

In future research, multi-target synergistic intervention may become a trend. During the occurrence and development of skin diseases, ROS are intertwined with other pathological processes caused by their accumulation (such as inflammation, microbial imbalance, etc.). Future therapeutic strategies will focus more on systematic regulation rather than just targeting ROS themselves. For example, simultaneous targeting of ROS-producing enzymes (like NOX inhibitors) and antioxidant defense systems (such Nrf2 activators) may be more successful than single intervention. Combining antioxidant therapy with immunomodulation, antibacterial, antifibrotic, and other strategies is expected to achieve synergistic effects.
